# MRI characterization of myocardial and microvascular injuries

**DOI:** 10.1186/1532-429X-15-S1-P4

**Published:** 2013-01-30

**Authors:** Loi Do, Mark W Wilson, Carol Stillson, Jessica K Murayama, Steven W Hetts, Maythem Saeed

**Affiliations:** 1 Radiology and Biomedical Imaging, University of California San Francisco, San Francisco, CA, USA

## Background

The NIH estimated that 7 million Americans suffer from coronary artery disease and ~500,000 patients die of heart attacks yearly. Jung et al found that the number of microemboli correlates with the size of myocardium at risk in patients with myocardial infarction [1]. Due to the population inhomogeneity it is difficult to grade and compare LV dysfunction in patients with acute microinfarct and large infarct caused by microemboli and LAD artery obstruction. The objectives of this study were to characterize and compare the effects of LAD occlusion/reperfusion and microvascular embolization on LV function using MRI.

## Methods

Pigs (24) were divided equally into 3 groups and subjected to: no-infarct (control), 90min of the LAD occlusion/reperfusion (large infarct) or microembolization (16 mm^3^, 40-120 μm). Three days after interventions, cine, perfusion and viability MRI (1.5T) were acquired to measure LV volumes, regional systolic wall thickening, regional perfusion and myocardial damage, respectively. Gd-DTPA was used for perfusion and viability imaging. At postmortem, the hearts were stained with TTC for postmortem analysis. A semi-automatic threshold method was used to measure infarcts.

## Results

**Figure 1:**
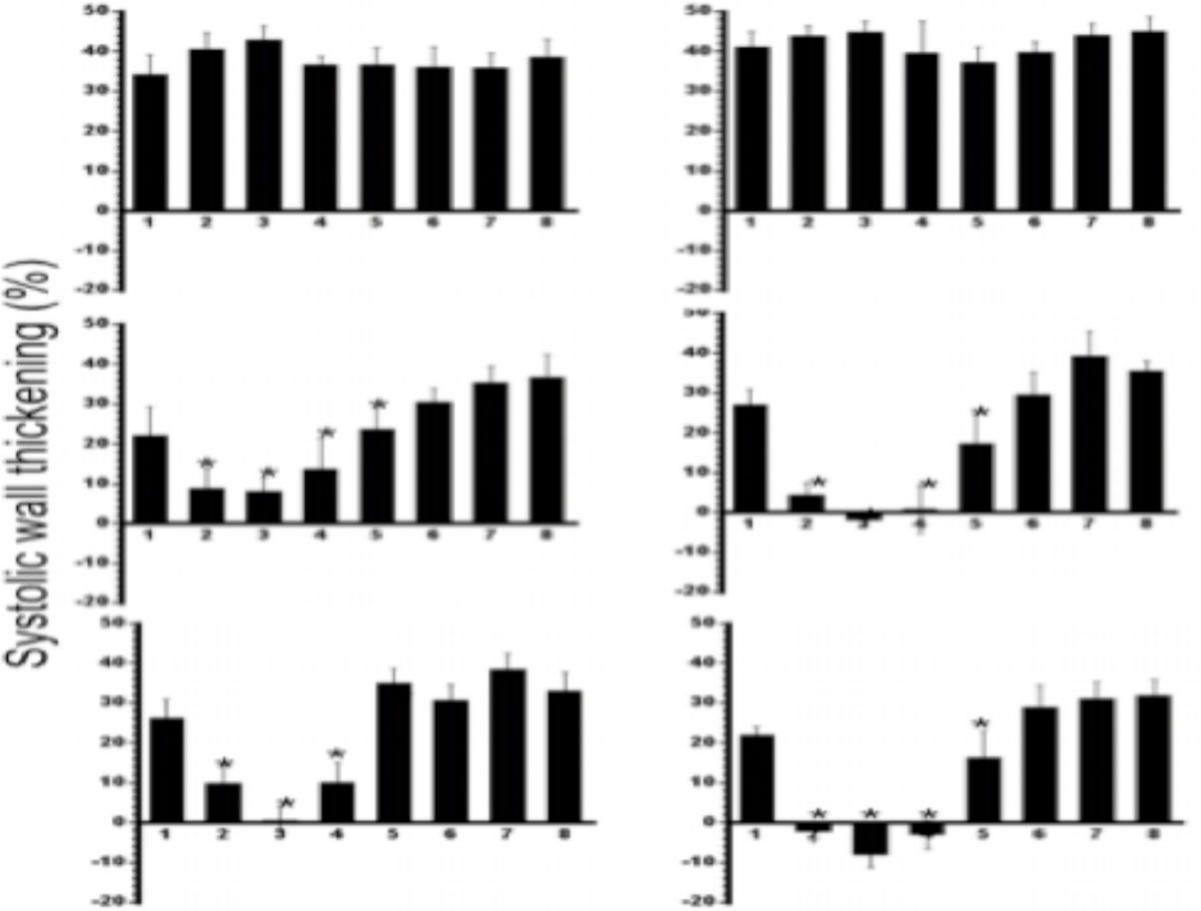
Systolic wall thickening obtained from 8 segments in two LV slices in control (top row), microembolized (middle row) and LAD occlusion (bottom row) animals. The segments with microinfarct and large infarct were graded as either hypokinetic, akinetic or dyskinetic compared to control animals. *P<0.01 compared with control groups.

Cine MR imaging demonstrated the difference in global left ventricular function between the groups. Animals subjected to coronary interventions showed comparable LV dysfunction compared with controls (Table 1). Furthermore, at the regional level, there was a significant difference in systolic wall thickening between control microembolized and large infarct animals (Figure 1).

**Table 1:** The effects of the interventions on global LV function compared with controls.

	EDV(ml)	ESV(ml)	Ejection fraction(%)
Control	77±3	35±2	52±1
Large infarct	75±2	44±1 *	41±1 +
Microinfarct	76±2	47±1 *	39±1 +

*P<0.05, +P<0.01

Both LAD microembolization and occlusion/reperfusion produced perfusion deficits in LAD territory compared to remote. Peak signal intensity and time to the peak were not significantly different between microembolized and occluded/reperfused animals. DE-MRI demonstrated hyperenhanced large infarct with microvascular obstruction in the core in 50% of the animals subjected to 90 min occlusion/reperfusion. Microinfarct caused by microemboli were patchy and randomly distributed in the the LAD territory. The size of large infarct was significantly greater than microinfarct on both MRI (12.6±1.2% LV mass and 6.5±0.6% P<0.01, respectively) and histochemical TTC staining (14.8±2.3% and 6.6±0.8% LV mass P<0.01, respectively). Control hearts revealed no infarct on either MRI or TTC.

## Conclusions

LV dysfunction in microembolized animals was disproportionally large and comparable to that produced by major coronary artery occlusion/reperfusion despite the substantial difference seen on contrast enhanced MRI. The decline in regional perfusion was also comparable between reperfused infarct and microinfarct. This study suggests that infarct size is not the sole factor for acute LV dysfunction in coronary microembolization.

## Funding

Departmental
